# Removal of Boron and Manganese Ions from Wet-Flue Gas Desulfurization Wastewater by Hybrid Chitosan-Zirconium Sorbent

**DOI:** 10.3390/polym12030635

**Published:** 2020-03-10

**Authors:** Joanna Kluczka

**Affiliations:** Department of Inorganic, Analytical Chemistry and Electrochemistry, Faculty of Chemistry, Silesian University of Technology, B. Krzywoustego 6, 44-100 Gliwice, Poland; joanna.kluczka@polsl.pl; Tel.: +48-32-237-1821

**Keywords:** boron adsorption, manganese ion adsorption, adsorption isotherms, column studies, desorption, biopolymer

## Abstract

Flue gas desulfurization (FGD) wastewater, after the alkaline precipitation and coagulation processes, often requires additional treatment in order to reduce the concentrations of boron and heavy metals below the required limits. In this study, we present an innovative and environmentally friendly method for boron and manganese removal that is based on a hybrid chitosan-zirconium hydrogel sorbent. The results from the batch adsorption experiment indicated that the uptake capacity for boron and manganese was equal to 1.61 mg/g and 0.75 mg/g, respectively, while the column study indicated that the total capacity of boron and manganese was equal to 1.89 mg/g and 0.102 mg/g, respectively. The very good applicability of the Langmuir isotherm at 25 °C suggested the monolayer coverage of the boron species onto the hybrid chitosan-zirconium hydrogel with a maximum adsorptive capacity of 2 mg/g. The amounts of boron and manganese in purified water could be decreased to less than 1 mg/dm^3^ and 0.05 mg/dm^3^, respectively, starting from the initial concentration of boron equal to 24.7 mg/dm^3^ and manganese equal to 3.0 mg/dm^3^ in FGD wastewater. Selective desorption of boron from the loaded bed was favorable when a NaOH solution was used, while manganese was preferentially eluted with a HCl solution. It is important to note that such an innovative method was investigated for the first time by testing borax recovery from wastewater in terms of an eco-technological perspective.

## 1. Introduction

The main source of energy in Poland is coal combusted in power plants. This leads to the generation of inconvenient by-products, namely flue gases [1]. One of the most commonly used flue gas desulfurization (FGD) technologies for scrubbing pollutants from power plant gas emissions is a wet scrubber system. In this process, many pollutants, including coal and limestone, end up in the circulating water that is partly discharged from the scrubber system. The stream contains contaminants from coal and limestone and is acidic and supersaturated with gypsum, with high concentrations of TDS (total dissolved solids), TSS (total suspended solids), heavy metals, chlorides, and occasionally, dissolved organic compounds [1,2]. Suspended solids are removed in the course of the alkaline precipitation and coagulation processes, followed by settling of the solids. FGD wastewater often requires additional treatment for the further elimination of boron and heavy metals [3].

Boron is a vital trace element that supports the growth of plants and is beneficial to the health of living organisms. Boron’s presence in the environment is a result of hydrothermal and volcanic activity, rock weathering, and desorbing from marine sediments [4]. Boron also enters the environment through anthropogenic activities, mainly as an effect of fossil fuel burning, boron mining and processing, glass production, as well as agricultural fires [5].

Manganese is also an essential element, affecting photosynthetic oxygen evolution in the chloroplasts of plants. In animals and humans, Mn plays an important role in iron metabolism and is required for proper brain function. Manganese exists both naturally and anthropogenically in the environment. It is released to the environment very slowly as a result of the chemical weathering of rocks and minerals. Manganese pollution is caused by anthropogenic activity, such as mining, crushing, and smelting of ores, as well as metallurgy, municipal wastewater discharges and sewage sludge, use of fungicides, and combustion of fossil fuels [5].

Boron and manganese compounds can have hazardous effects on plants, animals, and human beings when the supply of these elements is excessive. Therefore, their concentrations are limited in food and water. The recommended limit is set at 2.4 mg/dm^3^ for boron and at 0.4 mg/dm^3^ for manganese in drinking water (World Health Organization guidelines from 2011 and 2008, respectively). Boron is essential to plant growth, but may be toxic to crops when present in excessive concentrations in irrigation water. Sensitive plants show damage when irrigation water contains more than 670 μg/dm^3^, and even tolerant plants may be damaged when the concentration of boron exceeds 2000 μg/dm^3^. The recommended water quality criteria for long-term irrigation indicate 0.75 mg/dm^3^ for boron and 0.20 mg/dm^3^ for manganese, respectively [6]. In Poland, the limit of boron concentration equals 1 mg/dm^3^, while the limit of manganese concentration is 0.5 mg/dm^3^, both in natural water and in wastewaters discarded to the environment (Regulation of the Minister for Maritime Economy and Inland Navigation, 2019).

Considering the above-mentioned drawbacks, removing boron and manganese compounds from aqueous solutions is very important and necessary when they are found in high concentrations in water or wastewater.

Several different methods have been developed for removing heavy metals like manganese from solutions, including chemical precipitation, adsorption, ion exchange, reverse osmosis, ultrafiltration, and oxidation [7,8,9,10,11,12]. Among them, adsorption using natural sorbents is generally considered to be the most suitable method for wastewater treatment. Various sorbents have been studied so far for manganese ion removal: clay minerals, fish bone hydroxyapatite, fly ash, biomass, natural scolecite, as well as clinoptilolite from different regions [10,12,13,14]. Boron is not usually removed in the treatment of groundwater and fresh surface water used for drinking water. nor by standard sewage water treatment techniques. Among many methods of boron removal, adsorption is also the best choice. Sorbents studied in the literature include fly ashes (FAs) and other waste by-products [15,16], activated carbons, clays, and zeolites [17,18,19], as well as some oxides and hydroxides [20,21,22,23]. A very interesting group of sorbents is biosorbents [24,25,26,27,28,29], which can compete for efficiency with conventional adsorbents.

Chitosan is a biodegradable, non-toxic, bacteriostatic, and fungistatic bio-renewable polymer derived from chitin. Among these interesting properties, it also possesses adsorption abilities [30,31]. In its structure, chitosan has many functional groups such as N-acetyl, reactive hydroxyl, and amino groups, which can interact with particles present in aqueous solution or can be modified to generate various chitosan derivatives [32,33]. This is why chitosan is attractive as a potential sorbent in water and wastewater treatment [34]. Unfortunately, adsorption of boron species onto chitosan is characterized by limited capacity [35], probably because of the low yield of adsorption via physical force. Similarly, in the case of the adsorption of manganese ions on chitosan, the process follows a physical mechanism and has relatively small adsorptive capacity [36].

Modifying chitosan with various moieties and crosslinking with glutaraldehyde gave products more useful for the removal of Mn and B from wastewater. β-cyclodextrin-chitosan-graphene oxide composite material, carboxymethyl chitosan–hemicellulose resin, glycine modified chitosan resin, polyvinyl alcohol/chitosan, and magnetic nanocomposite of clinoptilolite/maghemite/chitosan/urea have been fabricated and successfully applied for the removal of manganese ions [37,38,39,40,41]. A method based on the ionic gelation of chitosan with various cations (Co^2+^, Ce^4+^, La^3+^, Ni^2+^, Fe^3+^) was also reported to turn the raw chitosan into an effective sorbent for borate [22,27,28,29,42].

In our earlier studies, it was demonstrated that amorphous zirconium (IV) dioxide, its blend with zeolite, and zirconium hydroxide could adsorb boron [19,21,43]. However, the adsorption of boric acid present in FGB wastewater on zirconium modified chitosan hydrogel has remained unrecognized until now. Furthermore, we have not found any information on the adsorption of manganese ions on hydrous zirconium dioxide or sorbents modified with zirconium hydroxide in the available literature. Accordingly, this paper intends (1) to investigate the performance of a zirconium modified chitosan hydrogel (Zr-CTS) in the adsorption of boron and manganese from a single aqueous solution in column mode, (2) to evaluate the possibility of the removal of both boron and manganese from wastewater in batch and column mode, and (3) to assess boron recovery from wastewater.

## 2. Materials and Methods

### 2.1. Reagents

Boric acid (H_3_BO_3_), manganese chloride (MnCl_2_), sodium hydroxide (NaOH), hydrochloric acid (HCl), nitric acid (HNO_3_), and glacial acetic acid (CH_3_COOH) were purchased from Avantor Performance Materials Poland S.A., Gliwice, Poland. All the reagents were analytically pure. Chitosan (CTS) (molecular weight of 600,000–800,000 g/mol) was purchased from Acros Organics (Geel, Belgium); the degree of deacetylation of CTS, as determined by means of a H^1^ NMR method, was equal to 97%. Zirconium chloride (ZrCl_4_) was purchased from Merck (Hohenbrunn, Germany). The wet-flue gas desulfurization (FGD) wastewater was obtained from the coal power plant with the composition shown in Table 1.

### 2.2. Analysis and Apparatus

The concentration of the elements was determined using ICP-OES (inductively coupled plasma optical emission spectroscopy) with a Varian 710-ES spectrometer (Varian, Mulgrave, Victoria, Australia). Calibration curves were prepared using standard solutions supplied by Merck KGaA (Darmstadt, Germany). Water was purified by a Millipore Elix 10 system.

A WU-4 universal shaker (Premed, Warsaw, Poland), WPE 120 electronic balance (Radwag, Radom, Poland), WPA 60/C analytical balance (Radwag, Radom, Poland), and pH-meter Basic 20+ (Crison Instruments, Barcelona, Spain) were used during the batch studies.

Peristaltic pump (Ismatec Reglo Digital, Cole-Parmer GmbH, Wertheim, Germany) was used for pumping the solution through the bed in the column studies.

### 2.3. Preparation of the Hybrid Chitosan-Zirconium Hydrogel Beads

Hybrid chitosan-zirconium sorbent was prepared according to the procedure described previously [43]. Briefly, a chitosan solution was prepared by dissolving 3 g of chitosan in 75 cm^3^ of 1 wt% acetic acid solution. Next, 3.83 g of ZrCl_4_ were added to the chitosan solution and sonicated for 30 minutes to obtain a well dispersed, homogenous solution. The chitosan-precursor solution was added dropwise, using a syringe with a thin needle (internal diameter of 0.9 mm), into a stirred 20 wt% aqueous NaOH solution. This resulted in the immediate coagulation and formation of beads. The as-formed chitosan beads were then filtered and washed with deionized water to remove traces of gelling solution. The prepared chitosan-zirconium sorbent was in the form of white hydrogel beads with an average diameter of about 4 mm. The total content of zirconium in the wet sorbent determined by the ICP-OES method was equal to 4.1 ± 0.1 wt%. The detailed characteristics of the hybrid chitosan-zirconium sorbent were described previously [43].

### 2.4. Adsorption and Desorption Methodology

#### 2.4.1. Batch Studies

A 10 cm^3^ amount of FGD wastewater of pH = 7.26 was added into a 50 cm^3^ conical flask containing from 0.05 to 2.00 g of the studied hybrid hydrogel beads (0.004–0.167 g of the dry form). The wastewater containing hydrogel beads was shaken at a mixing rate of 60 rpm in a mechanical shaker for an optimum time of 24 h (determined in the previous study [43]) at room temperature (25 ± 1°C). After filtration, the concentration of the elements in each solution was determined by means of the ICP-OES method. Each adsorption experiment was repeated three times to obtain an average value. The removal efficiency, *R* (%), and the adsorptive capacity of boron and manganese on the prepared beads, *q* (mg/g), were calculated using the Equations:

(1) $$R=\frac{\left(C_{0}-C\right)}{C_{0}}100$$

(2) $$q=\frac{\left(C_{0}-C\right)}{m}V_{0}$$

where *C*_0_ and *C* are the initial and final concentrations of boron or manganese in the solution (mg/dm^3^), respectively, *V*_0_ is the solution volume (dm^3^), and *m* is the dry mass of the sorbent (g).

Boron was successfully desorbed with 0.32 mol/dm^3^ NaOH solution according to the procedure described in the previous paper [43]. The desorption of manganese from the sorbent was examined as follows: 1.0 g of manganese loaded hydrogel beads and 10 cm^3^ of 0.001 mol/dm^3^ hydrochloric acid solution were mixed for 24 h at room temperature. The boron and manganese concentrations were determined using ICP-OES in the obtained aliquots.

The percentage of manganese desorption, *D* (%), was calculated using the Equation:

(3) $$D=\frac{q_{D}}{q}100$$

where *q_D_* is the amount of desorbed manganese per sorbent mass (mg/g).

To determine the margin of error, *ME* (%), a confidence interval of 95% was calculated for each set of samples using Microsoft Excel software.

#### 2.4.2. Column Studies

The column adsorption process was carried out in a 15 mm i.d. glass columns filled with the hybrid chitosan-zirconium sorbent in the form of hydrogel beads up to 150 mm. The feed solution (boric acid solution of initial concentration ~24 mg/dm^3^ at pH ~ 6 or manganese chloride solution of initial concentration ~4.5 mg/dm^3^ at pH ~ 6 or FGD wastewater at pH ~ 7.7) was passed through the column (drop-fed system) at a constant flow rate of 2 BV/h (bed volume per hour) using a peristaltic pump. The eluates from the column were collected into fractions of 5 cm^3^, and the concentration of the elements in each eluate was determined using the ICP-OES method. Each test was repeated twice.

The bed loaded with boron and manganese in the adsorption process was then used in the desorption studies. For this purpose, 20 cm^3^ of deionized water and the regenerating media: firstly 30 cm^3^ of 0.32 mol/dm^3^ NaOH (for boron desorption) and next 30 cm^3^ of 0.001 mol/dm^3^ HCl solution (for manganese desorption) were passed through the column at a constant flow rate of 1 BV/h at room temperature. Each test was repeated twice.

The breakthrough curves were obtained by plotting the fraction of the initial concentration of boron or manganese, *C*/*C*_0_, against the time of adsorption (min). The breakthrough time, *t_b_* (min), was set as the time when the effluent element concentration reached 4% of the influent concentration, while the exhaustion time, *t_total_* (min), was set as the time when the concentration of the effluent leveled with the influent concentration. Four percent of the concentration of boron in the effluent meant 1 mg/dm^3^, and this corresponded to the limit in natural water and in wastewaters discarded to the environment as set in Poland. The breakthrough adsorptive capacity of the element, *q_b_* (mg/g), was calculated using the Equation:

(4) $$q_{b}=\frac{V_{b}C_{0}}{m}$$

where *V_b_* is the eluate volume at the column breakthrough point (dm^3^), *C*_0_ is the initial concentration of the element at the column inlet (mg/dm^3^), and *m* is a sorbent mass (g). The total adsorptive capacity of the element (at the point of bed exhaustion in the column), *q_total_* (mg/g), was represented by the area under the adsorbed element concentration using the Equation given by Michaels [44]:

(5) $$q_{total}=\frac{F_{v}C_{0}}{m}\int_{0}^{t}\left(1-\frac{C}{C_{0}}\right)dt$$

which after integration is described by the Equation:

(6) $$q_{total}=\frac{F_{v}C_{0}t_{total}}{m}-\frac{F_{v}C_{0}}{m}\sum \frac{\frac{C_{i}}{C_{0}}+\frac{C_{i+1}}{C_{0}}}{2}\left(t_{i+1}-t_{i}\right)$$

where *F_v_* is the flow rate (dm^3^/min), *t_total_* is the time at the point of bed exhaustion (min), *C_i_* is the concentration of boron or manganese in the eluate *i* (mg/dm^3^), *C_i_*_+1_ is the concentration of boron or manganese in the eluate *i* + 1 (mg/dm^3^), *t_i_* is the time of collecting the eluate *i* (min), and *t_i_*_+1_ is the time of collecting the eluate *i* + 1 (min). The length of the mass transfer zone, *MTZ* (cm), was dependent on the bed height, *L* (cm), and the breakthrough and equilibrium point following the Equation [45]:

(7) $$MTZ=\left(\frac{t_{total}-t_{b}}{t_{total}}\right)L$$

The volume of effluent treated, *V_total_* (dm^3^), was calculated using the Equation:

(8) $$V_{total}=F_{v}t_{total}$$

## 3. Results and Discussion

### 3.1. Adsorption Isotherms

**Figure 1:**
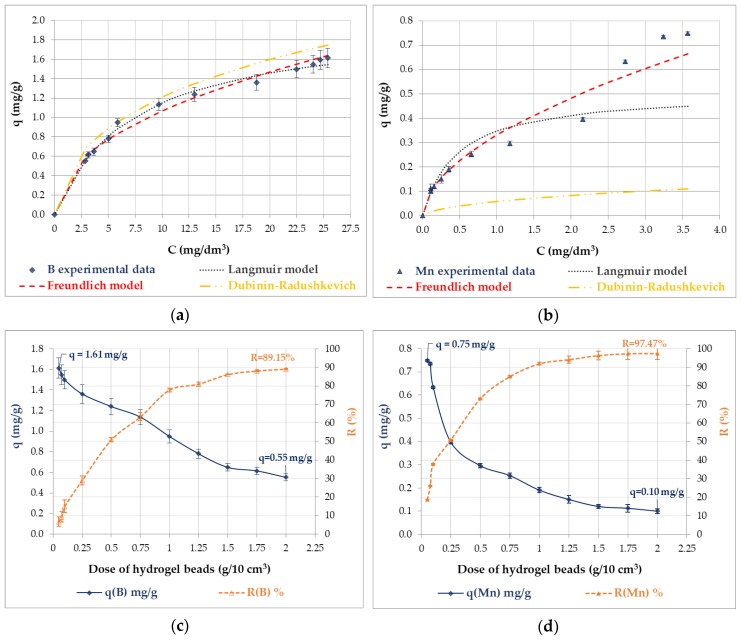
Boron (**a**) and manganese (**b**) adsorption isotherms on hybrid chitosan-zirconium hydrogel beads: experimental curves, Langmuir, Freundlich, and Dubinin–Radushkevich models, and the effect of hybrid chitosan-zirconium hydrogel bead amount on boron (**c**) and manganese (**d**) adsorption.

Boron and manganese adsorption isotherms and the effect of the amount of sorbent on boron and manganese adsorption are shown in Figure 1. The maximum adsorptive capacity of boron, *q*, was equal to 1.61 ± 0.1 at 25 °C, for a contact time of 24 h, and an initial boron concentration of 26.4 mg/dm^3^, while the adsorptive capacity of manganese was equal to 0.75 ± 0.01 at 25°C, for a contact time of 24 h, and an initial manganese concentration of 4.4 mg/dm^3^. Furthermore, a high removal efficiency was achieved, 89.2 and 97.5% for boron and manganese, respectively. In the equilibrium state, the content of boron and manganese in the hybrid chitosan-zirconium hydrogel beads (after dissolving three samples of thermally dried sorbent in concentrated HNO_3_) was equal to 1.645 ± 0.061 and 0.770 ± 0.005 mg/g, respectively, which agreed with the experimental adsorptive capacity calculated according to Equation (2).

To evaluate the mechanism of adsorption, three two-parameter models of isotherms describing the experimental data were used; Langmuir, Freundlich, and Dubinin–Radushkevich [46,47]. The most developed equations used to describe the adsorption equilibrium are mainly Langmuir and Freundlich; however, the Dubinin–Radushkevich isotherm is also often used to explain the equilibrium of adsorption by the theory of the volume filling of adsorbent micropores. The Langmuir model assumes that the adsorption occurs on surface sites where the energy is equal in each size, while the Freundlich model allows for several kinds of adsorption sites in the solid, each having different energies of adsorption.

The adsorption models used in this study and their equations in nonlinear and linear forms are presented in Table S1 (Supplementary Materials). The coefficients of the models were determined by linear regression. The degree of fit of the linear equations to the experimental points was assessed on the basis of the coefficient of determination, *R*^2^. The determined linear regression parameters were introduced into the model equations, and the fit was rated using the mean error, *ME* (%). Figure 1a,b show the fitting of the Langmuir, Freundlich, and Dubinin–Radushkevich adsorption models onto the experimental data, while the results of the boron and manganese adsorption modeling are presented in Table 2. As can be seen in Table S1 and Table 2, *q_m_* and B are the Langmuir parameters, *q_m_* is the adsorptive capacity (mg/g), expressed as the maximum amount of the element that can be adsorbed as a monolayer, and B is an equilibrium constant that corresponds to the adsorption energy (dm^3^/mg). The parameters *K_F_* ((mg/g) (dm^3^/mg)^1/*n*^) and *n*, resulting from the Freundlich model, correspond to the relative adsorptive capacity and the adsorption intensity of the adsorbent, respectively. According to Treybal [48], the suitability of using the Freundlich equation to describe the adsorption can be assessed by the constant *n*. If 1 < *n* < 10, the F equation is adequate for use. In the Dubinin–Radushkevich equation given in Table S1, *ε* is a Polanyi potential, which equals *RT*ln(1 + 1/c), while the *x_m_* parameter is the adsorptive capacity (mol/g), and the *k* parameter is a constant related to the adsorption energy (mol^2^/kJ^2^). The adsorption energy, *E* (kJ/mol), determining the energy required to transfer 1 mol of adsorbate species to the surface of the adsorbent from infinity in the bulk of the solution, is calculated as −(2*k*)^−0.5^. If the energy of adsorption is less than 20 kJ/mol, the adsorption is physical in nature due to the weak van der Waals force. The energy for chemisorption lies in the range between 40 and 800 kJ/mol [48].

As can be seen in Figure 1a and Table 2, the Langmuir model gave the best fit to the experimental data of boron adsorption on hybrid chitosan-zirconium hydrogel beads; the highest correlation coefficient, *R*^2^ = 0.992, and the smallest mean error, *ME* = 2.85%, were obtained at 25 °C. The very good applicability of the Langmuir isotherm at 25 °C suggested the monolayer coverage of boron molecules on the surface of hybrid chitosan-zirconium hydrogel where the active sites and energies were homogenously distributed [49,50]. The maximum adsorptive capacity was equal to 2 mg/g, which was in good agreement with the experimental value. A slightly worse fit was obtained for the Freundlich and Dubinin–Radushkevich models. On the basis of the Dubinin–Radushkevich model, it was possible to calculate a valuable parameter: the adsorption energy. The adsorption energy was below 20 kJ/mol and indicated a process having a physical nature. In the case of manganese adsorption, the best fit for the experimental data was given by the Freundlich model with the highest correlation coefficient (0.972) and the smallest mean error (9.60%) at 25 °C. The Freundlich model usually corresponds to the adsorption process on heterogeneous surfaces. As is observed in Table 2, the *K_F_* parameter was equal to 0.331, and the parameter *n* was in the range <1, 10>, indicating that the Freundlich model was adequate for describing the discussed process. The worst fit was obtained for the Dubinin–Radushkevich model, which was inadequate for describing manganese adsorption on hybrid chitosan-zirconium hydrogel beads (see Figure 1b).

Importantly, no presence of zirconium ions was detected in the solutions after adsorption, meaning that the modifier was not eluted from the sorbent and did not pollute the purified water.

### 3.2. Breakthrough Curves

**Figure 2:**
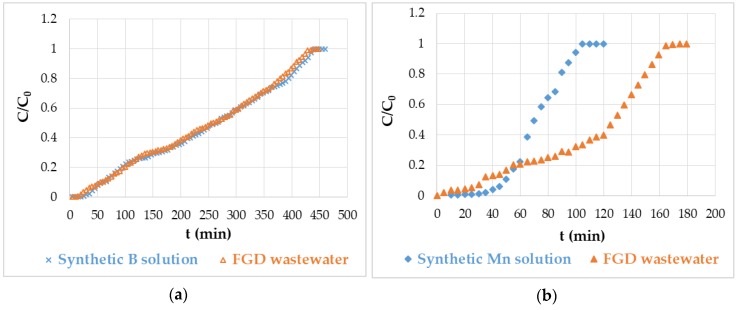
Boron (**a**) and manganese (**b**) breakthrough curves on hybrid chitosan-zirconium hydrogel beads.

The column performance in the removal of boron and manganese from synthetic solutions and real wastewater was analyzed using breakthrough curves. Figure 2 shows the boron and manganese breakthrough curves collected in both the synthetic solution and wastewater. The breakthrough curves of manganese assumed a characteristic S shape, while the breakthrough curves of boron indicated a slow process of saturating of the bed.

The adsorption calculations given in Table 3 show that the hybrid chitosan-zirconium was an effective sorbent for the removal of boron and manganese. Its total adsorptive capacity reached 1.89 mg/g for boron and 0.102 mg/g for manganese. It managed to pass ~450 cm^3^ of the solution or wastewater containing ~24 mg/dm^3^ of boron through 15 g of hydrogel till the exhaustion of the bed, out of which 40–50 cm^3^ of the adsorbate contained less than limit 1 mg/dm^3^ of boron. The analysis of zirconium ions revealed that they were not present in the effluents as a result of the purification of both the synthetic solution and wastewater. This meant that the modifier was not leached from the hydrogel beads to the effluent. The results indicated the possibility of purifying FGD wastewater below the concentration limit of boron (1 mg/dm^3^) and manganese (50 μg/dm^3^) imposed by government regulations, using the simplest system consisting even of one column.

The comparison of the total capacity of boron on the hybrid chitosan-zirconium bed with other sorbents in the column study is summarized in Table 4. It was clear that only boron selective resins (e.g., Lewatit MK, Diaion CRB, Dowex XUS, etc.) possessed slightly higher capacity for boron than the hybrid chitosan-zirconium hydrogel beads studied in this paper. It should be also emphasized that the proposed sorbent was an environmentally friendly biopolymer because of its biodegradability and non-toxicity.

### 3.3. Desorption

The regeneration of the loaded bed was favorably performed with NaOH and HCl. Zero-point-three-two moles per decimeter cubed of NaOH solution eluted 98.5% of boron from the saturated hybrid chitosan-zirconium hydrogel beads. In the eluate, the presence of manganese ions was not found, which proved the selective desorption of boron. In contrast, manganese was eluted with a 0.001 mol/dm^3^ HCl solution. The desorption percentage of manganese, *D*, was equal to 78.2%.

### 3.4. Purification of FGD Wastewater

**Figure 3:**
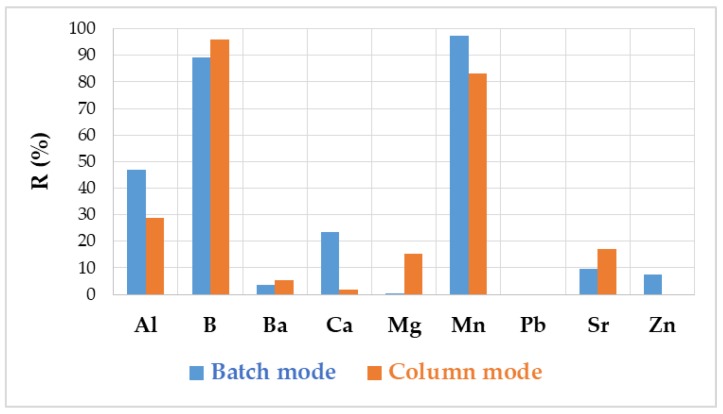
The effect of wastewater purification using hybrid chitosan-zirconium hydrogel beads on the effluent composition.

In the batch method, as a result of sewage FGD treatment using hybrid chitosan-zirconium hydrogel beads, the concentration of boron and manganese decreased by 89% and 97%, respectively, while the concentration of aluminum decreased by 46%, calcium by 23%, and other cations below 10%. At the breakthrough point of the column filled with hybrid chitosan-zirconium hydrogel beads, the resulting effluent contained 96% less boron and 83% less manganese. Other cations were not adsorbed much on the hydrogel (Figure 3).

## 4. Summary

Currently, ion exchange technique is used to remove boron from water and wastewater, most often in systems integrated with other separation techniques, such as precipitation, reverse osmosis, and electrodialysis [57,58]. In the wastewater after flue gas desulfurization, in the first stage, the wastewater was introduced into the alkaline precipitation unit, in which boron was removed only to a small extent. In the second stage, boron retention on boron selective resins (BSR) occurred. Next, the adsorbed boron was removed from the resin with sulfuric acid, and the resulting mixture of acids was separated: sulfuric acid was recycled while orthoboric acid was concentrated and crystallized [58]. Despite many advantages, the method had a serious drawback, namely high investment and operating costs [3,57]. In this and earlier studies [43], it was found that boron could be efficiently removed by hybrid chitosan-zirconium hydrogel beads over a wide pH range, which made this sorbent competitive with BSRs, e.g., Lewatit MK 51, Purolite S 108, and Amberlite IRA 743 [51,53].

**Figure 4:**
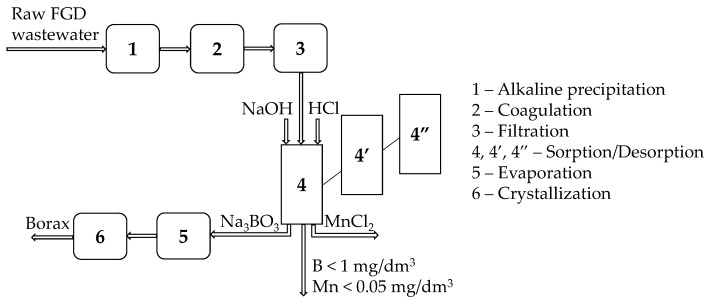
Block process flow sheet of the removal and recovery of boron.

Given the results obtained, a new strategy for boron removal and recovery was proposed (Figure 4). The system consisted of two or three columns working alternately (when the first one was working, the second one was regenerated with NaOH or HCl solution). Columns were filled with granules of hybrid chitosan-zirconium hydrogel. After passing through the column, wastewater could be discharged to sewage systems (B < 1 mg/dm^3^ and Mn < 0.05 mg/dm^3^). The solution after desorption (NaOH and Na_3_BO_3_) was concentrated and then crystallized to borax. Manganese was recovered in the form of MnCl_2_ solution. The investment and operating costs would be significantly lower than for current technology because the solution after desorption did not require the use of additional separation operations, as was the case after BSR regeneration (sulfuric acid used for boron desorption must be separated from boric acid before concentration and crystallization), and boron was recovered as borax.

## Figures and Tables

**Table 1:** Soluble constituents, pH reaction, and electrolytic conductance of the tested flue gas desulfurization (FGD) wastewater.

**Al (mg/dm^3^)**	**B (mg/dm^3^)**	**Ba (mg/dm^3^)**	**Ca (g/dm^3^)**	**Cl (g/dm^3^)**	**F (mg/dm^3^)**	**K (mg/dm^3^)**	**Li (mg/dm^3^)**	**Mg (mg/dm^3^)**
0.130	26.5	21.2	1.10	2.16	1.43	74.0	0.797	394
**Mn (mg/dm^3^)**	**Na (mg/dm^3^)**	**Pb (mg/dm^3^)**	**S (mg/dm^3^)**	**Sr (mg/dm^3^)**	**Zn (mg/dm^3^)**	COD * (mgO_2_/dm^3^)	**pH**	**Electrolytic Conductance (mS/cm)**
4.64	599	0.258	170	19.6	8.33	128	7.26	51

* COD—chemical oxygen demand.

**Table 2:** Parameters of the Langmuir, Freundlich, and Dubinin–Radushkevich isotherms on hybrid chitosan-zirconium hydrogel beads at 25 ± 1 °C.

Isotherm Model	Element	Parameters of the Isotherm Models, *q*(Mn)_exp_ = 0.75 ± 0.01 mg/g (for *C*_0_ = 4.39 mg/dm^3^), *q*(B)_exp_ = 1.61 ± 0.1 mg/g (for *C*_0_ = 26.4 mg/dm^3^)			
		*q_m_* (mg/g)	B (dm^3^/mg)	*R* ^2^	*ME* (%)
Langmuir	Mn	0.508	2.16	0.941	19.2
	B	2.00	0.136	0.992	2.85
		*K_F_* ((mg/g) (dm^3^/mg)^1/n^)	*n*	*R* ^2^	*ME* (%)
Freundlich	Mn	0.331	1.82	0.972	9.60
	B	0.369	2.17	0.977	4.35
		*X_m_* (mg/g)	*E* (kJ/mol)	*R* ^2^	*ME* (%)
Dubinin–Radushkevich	Mn	5.98	−11.2	0.964	82.9
	B	5.23	−10.0	0.985	8.96

**Table 3:** Evaluation of the column mode performance of the hybrid chitosan-zirconium hydrogel beads for boron and manganese ions’ removal.

Element	Influent	*C*_0_ mg/dm^3^	*V_b_* (cm^3^)	*C_b_* (mg/dm^3^)	*q_b_* (mg/g)	*V_total_* (cm^3^)	*q_total_* (mg/g)	*MTZ* (cm)	*q_expt_* (mg/g)
B	Synthetic solution	23.43	40	0.991	0.306	450	1.87	13.7	1.986
B	FGD wastewater	24.67	30	0.998	0.238	445	1.89	14.0	1.92
Mn	Synthetic solution	4.67	50	0.505	0.076	110	0.108	8.2	0.105
Mn	FGD wastewater	3.021	50	0.507	0.049	175	0.102	10.7	0.106

**Table 4:** Summary of selected column studies on boron removal conducted on commercial and new sorbents.

Sorbent	Initial Concentration	Breakthrough Capacity	Adsorption Uptake	Exhaustion Capacity	Sorbent Mass/ Volume	Ref.
Lewatit MK 51	33.9 mg/dm^3^	1.56 mg/cm^3^	99%	2.48 mg/cm^3^	2 cm^3^	[51]
Diaion CRB 03	33.9 mg/dm^3^	2.36 mg/cm^3^	87%	4.08 mg/cm^3^	2 cm^3^	[51]
Ca-Alginate gel beads	25 mg/dm^3^	-	43%	2.07 mg/g	1.44 g	[52]
Dowex XUS 43594,00	8.5-13 mg/dm^3^	2.5 mg/g	81%	4.8 mg/g	3 cm^3^	[53]
Fly ash agglomerates	100 mg/dm^3^	-	-	0.897 mg/g	18 g	[54]
Chitosan/Fe(OH)_3_	4.2 mg/dm^3^	-	29%	0.08 mg/g	7.2 g	[55]
Curcumin-AC	890 mg/dm^3^	-	99%	1.70 mg/g	227 g	[56]
Chitosan/Zr(OH)_4_ hydrogel beads	24.7 mg/dm^3^	0.238 mg/g	89%	1.89 mg/g	15 g	This study

## Supplementary Materials

The following are available online at https://www.mdpi.com/2073-4360/12/3/635/s1. Table S1: Langmuir, Freundlich, and Dubinin–Radushkevich models of the adsorption isotherms [46,47].
