# Supplementary material for: Removal of Boron and Manganese Ions from Wet-Flue Gas Desulfurization Wastewater by Hybrid Chitosan-Zirconium Sorbent
Source: Polymers (Basel). 2020 Mar 10;12(3):635. doi: 10.3390/polym12030635 (PMC7183067; doi:10.3390/polym12030635)
Supplement: Supplementary file 1 [file polymers-12-00635-s001.pdf]

Supplementary Data

Article

Removal of Boron and Manganese Ions from the Wet-Flue Gas Desulfurization Wastewater by Hybrid Chitosan-Zirconium Sorbent

Joanna Kluczka

Department of Inorganic, Analytical Chemistry and Electrochemistry, Faculty of Chemistry, Silesian University of Technology, B. Krzywoustego 6, 44-100 Gliwice, Poland; joanna.kluczka@polsl.pl;
Tel.: +48-32-237-1821

Table S1. Langmuir, Freundlich and Dubinin-Radushkevich models of adsorption isotherms [46,47].

Model	Nonlinear equation	Linear equation	Plot
L	$q_e = q_m \times \frac{B \times C_e}{1 + B \times C_e}$	$\frac{1}{q_e} = \frac{1}{q_m \times B \times C_e} + \frac{1}{q_m}$	$\frac{1}{q_e} vs \frac{1}{C_e}$
F	$q_e = K_F \times\ (C_e)^{1/n}$	$log(q_e) = log(k_F) + \frac{1}{n} log(C_e)$	$log(q_e)\ vs\ log(C_e)$
D–R	$q_e = (X_m)\ exp(-k \times \varepsilon^2)$	$ln(q_e) = ln(X_m) - k \times \varepsilon^2$	$ln(q_e)\ vs\ \varepsilon^2$
